# The association of migration and ethnicity with use of the Improving Access to Psychological Treatment (IAPT) programme: a general population cohort study

**DOI:** 10.1007/s00127-021-02035-7

**Published:** 2021-02-16

**Authors:** Vishal Bhavsar, Sohail Jannesari, Philip McGuire, James H. MacCabe, Jayati Das-Munshi, Dinesh Bhugra, Sarah Dorrington, June S. L. Brown, Matthew H. Hotopf, Stephani L. Hatch

**Affiliations:** 1 Section of Women’s Mental Health, Department of Health Services and Population Research, King’s College London, Institute of Psychiatry, Psychology and Neuroscience, 16 De Crespigny Park Road, London, SE5 8AZ UK; 2 NIHR Specialist Mental Health Biomedical Research Centre (BRC) At South London and Maudsley NHS Foundation Trust, London, SE5 8AF UK; 3 Department of Psychosis Studies, King’s College London, Institute of Psychiatry, Psychology and Neuroscience, 16 De Crespigny Park Road, London, SE5 8AZ UK; 4 Department of Psychological Medicine, King’s College London, Institute of Psychiatry, Psychology and Neuroscience, 16 De Crespigny Park Road, London, SE5 8AZ UK; 5 Department of Affective Disorders, King’s College London, Institute of Psychiatry, Psychology and Neuroscience, 16 De Crespigny Park Road, London, SE5 8AZ UK; 6 Department of Psychology, King’s College London, Institute of Psychiatry, Psychology and Neuroscience, 16 De Crespigny Park Road, London, SE5 8AZ UK; 7 Economic and Social Research Council (ESRC) Centre for Society and Mental Health, King’s College London, London, UK

**Keywords:** Migration, Ethnicity, Health inequalities, Psychological treatment, Common mental disorders

## Abstract

**Background:**

Common mental disorders (CMD), such as depression and anxiety, are an important cause of morbidity, economic burden and public mental health need. The UK Improving Access to Psychological Therapies (IAPT) programme is a national effort to reduce the burden and impact of CMD, available since 2008.

**Aims:**

To examine ethnic and migration-related differences in use of IAPT-based psychological treatment using a novel epidemiological dataset with linkage to de-identified IAPT records.

**Method:**

Data from a psychiatric morbidity survey of two South East London boroughs (2008–2010) were individually-linked to data on IAPT services serving those boroughs. We used Poisson regression to estimate association between ethnicity and migration status (including years of UK residence), with rate of subsequent use of psychological treatment.

**Results:**

The rate of psychological treatment use was 14.4 cases per thousand person years [cases/1000 pyrs, 95% confidence intervals (95% CI) 12.4, 16.7]. There was strong statistical evidence that compared to non-migrants, migrants residing in the UK for less than 10 years were less likely to use psychological treatment after adjustment for probable sociodemographic predictors of need, life adversity, and physical/psychiatric morbidity at baseline [rate ratio (RR) 0.4 (95% CI 0.20, 0.75]. This difference was not explained by migration for asylum/political reasons, or English language proficiency, and was evident for both self- and GP referrals.

**Conclusions:**

Lower use of IAPT among recent migrants is unexplained by sociodemographics, adversity, and baseline morbidity. Further research should focus on other individual-level and societal barriers to psychological treatment use among recent migrants to the UK, including in categories of intersecting migration and ethnicity.

**Supplementary information:**

The online version contains supplementary material available at 10.1007/s00127-021-02035-7.

## Introduction

Reducing the burden and impact of common mental disorders (CMD), including depression and anxiety, is a key challenge for population health globally [35]. In the UK, the Improving Access to Psychological Treatments (IAPT) programme, established in 2008, aims to address this challenge by improving accessibility of evidence-based psychological treatment for CMD [22]. UK Department of Health guidance states the importance of ensuring *equity* in IAPT treatment, that is, limiting avoidable, unfair, or remediable differences in access across subgroups within the population, including those based on age, gender, and ethnicity [13]. This guidance, therefore, allows for receipt of IAPT treatment triggered either by referrals from individuals themselves (self-referrals by website or telephone) or via general practitioners (GPs); self-referrals are proposed to improve the equity of service use among those affected by disparities, including Black, Asian, and Minority ethnic (BAME) communities [3, 14]. Many individuals and families from BAME communities have a history of recent or historic migration—in the 2011 census, 93% of White individuals reported being born in the UK, compared to 60% of self-described Black Caribbeans, 41% of Black Africans, and 46% of Asians [30]. Migrants may arrive in a host country without proficiency in the host country’s language. Proficiency in the majority language is associated with access to, and quality of, mental health services among migrants [2, 27, 32].

There has been limited research on equity of psychological treatment use among migrants, including people migrating to seek asylum/fleeing war. Migrants could have lower levels of awareness of the existence/availability of psychological treatment, particularly where those services are more recently established such as IAPT, compared to services of longer standing, such as secondary mental health services. More recent migrants also appear to have lower levels of GP registration, which is necessary to use IAPT-based psychological treatment via GP referral [33]. We have previously reported that survey respondents in South East London who had migrated to the UK for asylum or political reasons were likelier to report need for mental health services, compared to people migrating for economic/educational reasons [17]. This analysis was based on a self-reported mental health service use outcome, rather than use of psychological treatment specifically. There is also limited research on disparities in psychological treatment use between different ethnic groups in the UK, although there are well-known and persisting disparities in *secondary* mental health service use between ethnic groups [7]. We previously examined equity of access to IAPT by comparing IAPT-treated patients drawn from a case register, with survey data from the catchment general population, with and without CMD. In this study, while the proportion of Black Caribbean individuals in the IAPT group was similar to that of the general population group, larger differences were found among people of Black African ethnicity—16.1% of the catchment population was of Black African ethnic group, while Black African service users made up 6% of the IAPT-treated group [9]. However, previous research on disparities in IAPT use has not accounted for factors influencing use of psychological treatment. Accounting for underlying differences in the distribution of CMD, as well as poor physical health [16], as predictors of IAPT access requires general population data which has rarely been available to investigators of data on IAPT cases. Exposure to adversity, including physical/sexual abuse during childhood and adverse life events in adulthood, increase use psychological treatment [12], but studies of disparities in psychological treatment use have not accounted for this. Hepgul and colleagues estimated a high prevalence of alcohol use disorders in IAPT cases, but data on alcohol use in the catchment general population was unavailable in this study, limiting assessment of alcohol use as an explanation for differences [21]. Finally, studies on differences in IAPT use have been unable to account for use of secondary mental health services for CMD as an alternative, or competing, outcome [21].

We used novel epidemiological linked data to examine incidence of psychological treatment use (IAPT) in a geographically-defined ethnically diverse urban catchment, extending previous work and accounting for relevant predictors of psychological treatment use. We aimed to:

- Assess ethnic and migration-related differences in incidence of IAPT-based psychological treatment use,
- Evaluate alternative explanations for any associations, such as predictors of psychological treatment need and use of secondary mental health services for CMD, exploring the association in self-referral and GP-referrals separately, and
- Examine whether differences in psychological treatment use are explained by reasons for migration, or by English proficiency. Because of inherent collinearity between English proficiency and being born in the UK, and between being born in the UK and reasons for migration, this aim was examined in migrants only.

## Methods

### Details of sampling

We did a cohort study using a community health survey, with prospectively collected outcomes drawn from individual linkage to two related mental healthcare provider databases. The South East London Community Health Study, SELCoH [20] is a representative household survey whose first wave (SELCoH-1) took place in 2008–2010. The survey used random household sampling to identify a representative sample of adults aged 16 years and older living in Lambeth and Southwark. Sampling was clustered by household, with all adults living in selected households invited to participate. Full details of the study, sampling methods, and representativeness are published [19]. Among 1698 participants surveyed, 86% (1455) gave permission for linkage to mental health records, where available.

### Measurement of outcome

The outcome was use of IAPT-based psychological treatment. Data on use of IAPT-based psychological treatment and non-IAPT mental health services among SELCoH participants were derived from two databases within the National Institute of Health Research Maudsley Biomedical Research Centre—these were the Clinical Record Interactive Search (CRIS) database and the CRIS-IAPT database. The South London and Maudsley NHS Foundation Trust (SLaM) is the sole provider of mental healthcare, including IAPT services, in the two boroughs of South London that were surveyed in SELCoH-1, covering a catchment population of approximately 0.62 million. The Trust has used a single electronic system, IAPTUS, to hold clinical data on IAPT-based treatment, including structured fields for referral date, referral source, treatment episodes and outcome since 2008. De-identified IAPTUS data are stored in the CRIS-IAPT database. Linkage of SELCoH-1 to CRIS-IAPT was carried out by an independent in-house informatics team, the SLaM Clinical Data Linkage Service (CDLS), and used personal identifiers (name, date of birth, NHS number, postcode and gender) to probabilistically link survey data with matching electronic health records. Following linkage, data on SELCoH-1 participants who had consented to record linkage were then scrutinised in IAPT-CRIS for date of referral. This linked information was used to derive time from SELCoH-1 interview to use of psychological treatment (from IAPT-CRIS). To gather data on use of non-IAPT mental health services, we used a previously described linkage of SELCoH-1 records to the CRIS database, a separate database of de-identified clinical data on referrals to inpatient and community mental health services covering the same catchment area [5].

### Measures

#### Data on ethnicity and migration

Self-described ethnicity was collected at SELCoH-1 according to census categories set by the UK Office for National Statistics (ONS) and collapsed into a five-category variable comprising White, Black African, Black Caribbean, South Asian (comprising Indian, Pakistani, and Bangladeshi self-described ethnicity), and Other (Chinese, Black other, mixed, and all other) groups. For migration status, we collected information on whether the participant was born in the UK, and if not, how long ago, in years, they migrated to the UK. We classified this information into four categories: (1) born in the UK, (2) not born in the UK and living the UK for more than 20 years, (3) not born in the UK and living in the UK for 10–20 years, and (4) not born in the UK and living in the UK for less than 10 years. The number of categories was determined to limit very small numbers of outcomes occurring in each category, and was in line with previous work using these data [19].

#### Sociodemographic information

We grouped age into categories for participants aged 17–35, 36–54, and 55 and older. Highest educational attainment was categorised into “no qualifications”, “GCSE/O-level”, “A-Level”, and “degree level and above”, incorporating their non-UK equivalents where possible. Employment status was categorised into employed, student, unemployed and other (including temporary sick or permanent sick/disabled, retired or looking after the home with children). Relationship status was classified into: single, married/cohabiting, divorced/separated and widowed. Participants were classified by borough of residence at interview (Lambeth or Southwark).

#### Lifetime adverse life events and childhood abuse

Physical abuse, and sexual abuse, before the age of 16 were collected as separate items, and combined into a single binary indicator for the experience of any childhood physical/sexual abuse. Information on adverse life events was based on separate items for the following adverse life events, occurring since aged 16: witnessed violence, being exposed to a war zone, being victim to a crime, injury with a weapon, being physically/sexually attacked, separation from a partner, death of a loved one, serious accident/injury, and homelessness.

#### Common mental disorders (CMD), poor physical functioning, and GP registration

The Clinical Interview Schedule (Revised, CIS-R) [23], was used to assess symptoms of common mental disorders (CMD), assigning a numerical score categorised into three groups: 0–11 (no CMD), and 12–17 for mild-moderate CMD, and 18 and above, reflecting the presence of CMD warranting treatment. In line with previous work, we ascertained poor physical functioning using the physical component of the SF-12 [36], generating a binary indicator for respondents falling into the lowest quartile of scores on this scale. Because of the presumed association between GP registration and referral to IAPT via GP, we also used self-reported information on GP registration at interview.

#### Drug use and hazardous alcohol use

Separate items on whether or not respondents had used, in the past year, cannabis, crack, cocaine, ecstasy, LSD, and heroin were used to derive a single binary variable for any illicit drug use in the previous year. Hazardous alcohol use was measured by applying a cut-off of 8 on the AUDIT questionnaire [31].

#### Migration for asylum/political reasons and language proficiency

Free text responses to a question on reasons for migration were classified into migrating for asylum or political reasons, for work or study, for family reasons, for a better life, or for other reasons, based on previous work [17]. Respondents were also asked if English was their first language, generating a binary variable indicating English proficiency. We also used information on the continent from which migrants emigrated to the UK.

### Analyses

We carried out analyses in Stata 14 and accounted for survey non-response and household clustering [35]. The outcome (use of psychological treatment) was described stratified by each analysed covariate. We reported *p*-values to 2 decimal places, and other quantities to 1 decimal place [1]. To calculate rates, data were set for survival analysis specifying the SELCoH-1 interview date as the entry point, and first use of IAPT-based psychological treatment as the event of interest. Conclusion of follow-up time was defined as removal from the population at risk, either due to the outcome (use of psychological treatment), due to coming to the end of follow-up time (November 2018) or due to being untraceable or dead at date of follow-up interview. Rates were estimated in the overall sample, and by each included covariate. For descriptive purposes, we estimated unadjusted rate ratios for each covariate, using Poisson regression with Wald *p*-values. For ordered categorical covariates, we assessed linearity using likelihood ratio tests comparing fit of linear and indicator terms.

We used multivariable Poisson regression models to examine our aims, adjusting for a comprehensive range of explanatory variables. First, to estimate unadjusted associations for migration status and ethnic group with use of psychological treatment, we included both of these variables in the same model. Secondly to examine the impact of adjusting for distinct groups of explanatory variables on our results, we estimated partially-adjusted regression models, grouping adjustment variables into (a) calendar period and basic demographic and socioeconomic variables (age, gender, borough of residence, employment, educational attainment, and marital status, model 1), (b) markers of psychological treatment need (CMD and poor physical functioning, model 2), (c) adversity (childhood abuse and adverse life events, model 3), (d) drug use and hazardous alcohol use (model 4), and (e) GP registration at interview (model 5). Based on limited attenuation of our estimates, we present model estimates from the unadjusted model and the fully adjusted model (model 5), and report partially adjusted model estimates in the Supplement Table S2. To account for use of non-IAPT based mental health services, we estimated our fully adjusted model in competing risks regressions with referral to secondary mental health services as a competing outcome. Although based on small numbers of outcomes, we also estimated final models for self-referral rate and GP-referral rate separately, by coding the other outcome as censored (Table S3).

To examine the third aim, we estimated the association of years of residence with use of psychological treatment, adjusting for migrating for asylum/political reasons and English proficiency (in addition to adjustment variables described above). Owing to inherent collinearity between being UK-born and English proficiency, and between being UK-born and not migrating for asylum, this aim was examined in migrants only. We used likelihood ratio tests comparing linear and indicator forms of the years of residence variable to assess statistical evidence for a trend in associations with increasing years of residence. We also described continent of migration and reasons for migration by ethnic group, to further describe the sample (Table S4).

## Results

### Sample characteristics

Of 1698 SELCoH-1 survey participants, 1455 consented to data linkage to electronic health records (described in Table 1). Among this group, a weighted percentage of 62.9% (912) were born in the UK, 10.6% (164) were migrants residing in the UK for longer than 20 years, 9.3% (135) were migrants residing in the UK for 10–20 years, and 17.1% (244) were migrants residing in the UK for less than 10 years, at interview. 63.7% (934) of the sample were of White ethnicity, with Black African then next most prevalent ethnic category (13.1%, 187 individuals) followed by other (11.8%, 168), Black Caribbean (7.9%, 116), and the South Asian group (3.4%, 48). The youngest age band (16–34) was most prevalent in the sample, at 45.5% (622). Females (52.3%, 822), were more prevalent in the sample than males (47.7%, 633). A very high proportion of respondents, 96.1% (1402), were registered with a GP.

**Table 1:** Description of 1455 survey participants consenting to record linkage to data on psychological treatment

	Total (% of sample)	Referral to IAPT	Rate per 1000 person-years (95% CI)		Rate ratio (RR, 95% CI)	Wald test *p*-value for RR
Migration status						
Born in the UK	912 (62.9)	126	19.0 (16.0, 22.6)		Reference	
Not born in UK: years of UK residence						
> 20	164 (10.6)	21	17.0 (11.1, 26.0)		0.91 (0.56, 1.47)	
10–20	135 (9.3)	20	21.1 (13.6, 32.8)		1.11 (0.69, 1.78)	
< 10	244 (17.1)	12	7.1 (4.0, 12.5)		0.36 (0.20, 0.66)	< 0.01
Ethnicity						
White	934 (63.7)	121	17.8 (14.9, 21.3)		Reference	
Black Caribbean	116 (7.9)	11	12.5 (6.9, 22.5)		0.72 (0.39, 1.33)	
Black African	187 (13.1)	16	12.2 (7.5, 20.0)		0.68 (0.41, 1.14)	
South Asian	48 (3.4)	6	16.4 (7.4, 36.5)		0.91 (0.43, 1.92)	
Other	168 (11.8)	25	21.7 (14.6, 32.1)		1.20 (0.75, 1.93)	0.39
Missing	2 (0.1)	0	0		–	
Year of interview						
2008	118 (8.1)	20	20.2 (13.0, 31.2)		Reference	
2009	663 (45.5)	74	14.8 (11.7, 18.5)		0.73 (0.44, 1.22)	
2010	674 (46.4)	85	18.9 (15.3, 23.4)		0.94 (0.58, 1.55)	0.24
Calendar period of observation						
2008–2009	–	7	14.3 (6.8, 30.1)		Reference	
2010–2011	–	31	12.1 (8.5, 17.3)		0.80 (0.35, 1.81)	
2012–2013	–	57	22.2 (17.1, 28.7)		1.49 (0.67, 3.29)	
2014–2015	–	42	17.4 (12.8, 23.5)		1.12 (0.50, 2.50)	
2016–2018	–	42	17.0 (12.5, 22.9)		1.12 (0.50, 2.50)	0.11
Age at interview						
16–34	622 (45.5)	73	16.58 (13.2, 20.9)		Reference	
35–54	530 (35.5)	79	20.40 (16.4, 25.4)		1.25 (0.91, 1.72)	
55–	303 (19.0)	27	12.09 (8.3, 17.6)		0.74 (0.46, 1.18)	0.06
Gender						
Male	633 (47.7)	54	11.66 (8.9, 15.2)		Reference	
Female	822 (52.3)	125	21.26 (17.8, 25.3)		1.82 (1.32, 2.51)	< 0.01
Borough of residence						
Southwark	732 (50.4)	102	20.1 (16.6, 24.4)		Reference	
Lambeth	723 (49.6)	77	14.2 (11.3, 17.7)		0.70 (0.52, 0.95)	0.02
Employment						
Employed	784 (54.1)	84	14.7 (11.9, 18.2)		Reference	
Students	209 (15.7)	25	16.5 (11.1, 24.4)		1.13 (0.72, 1.77)	
Unemployment	144 (10.0)	6	26.8 (19.3, 39.4)		1.84 (1.15, 2.92)	
Other	312 (19.8)	44	19.4 (14.4, 26.0)		1.35 (0.93, 1.96)	0.06
Missing	6 (0.4)	0	0		–	
Educational attainment						
No qualifications	196 (12.7)	23	16.1 (10.7, 24.3)		Reference	
GCSE	299 (20.7)	43	20.1 (14.9, 27.1)		1.22 (0.74, 2.00)	
A level	354 (24.9)	48	19.0 (14.4, 25.3)		1.14 (0.69, 1.88)	
Degree level or above	591 (40.7)	63	14.6 (11.4, 18.7)		0.87 (0.53, 1.44)	0.30
Missing	15 (0.9)	2	16.8 (4.2, 67.2)		–	
Marital status						
Single	558 (40.2)	71	18.0 (14.3, 22.7)		Reference	
Married/cohabiting	692 (46.8)	76	14.9 (11.9, 18.6)		0.81 (0.58, 1.15)	
Divorced/separate	161 (10.3)	26	22.8 (15.5, 33.5)		1.29 (0.83, 2.01)	
Widowed	44 (2.6)	6	18.9 (8.5, 42.0)		1.09 (0.49, 2.46)	0.23
Number of lifetime adverse life events^a^						
0–2	750 (51.5)	80	14.7 (11.8, 18.2)	Reference		
3–5	599 (41.2)	78	18.0 (14.4, 22.5)	1.26 (0.92, 1.73)		
6–8	106 (7.3)	21	29.2 (19.1, 44.8)	1.98 (1.23, 3.20)		0.02^e^
Childhood abuse						
No	1058 (72.4)	116	15.0 (12.5, 19.0)	Reference		
Yes	384 (26.8)	61	22.6 (17.6, 29.1)	1.49 (1.09, 2.04)		0.01
Missing	13 (0.8)	2	22.0 (5.5, 87.8)	–		
Common mental disorder (CIS-R score)^b^						
0–12	1102 (76.2)	106	13.0 (1.07, 15.7)	Reference		
12–17	174 (11.9)	32	27.2 (19.3, 38.5)	2.09 (1.40, 3.12)		
18 and above	176 (11.7)	41	35.9 (26.4, 48.7)	2.80 (1.94, 4.04)		< 0.01^f^
Missing	3 (0.2)	0	–	–		
Drug use^c^						
No	1132 (76.6)	128	15.5 (13.0, 18.4)	Reference		
Yes	319 (23.1)	50	22.7 (17.2, 30.0)	1.46 (1.03, 2.07)		0.03
Missing	4 (0.3)	1	–			
Hazardous alcohol use^d^						
No	1146 (77.9)	143	17.2 (14.6, 20.3)	Reference		
Yes	300 (21.5)	35	16.3 (11.7, 22.7)	0.95 (0.65, 1.39)		0.79
Missing	9 (0.6)	1	–			
Poor physical functioning						
No	1187 (83.1)	127	14.3 (12.0, 17.0)	Reference		< 0.01
Yes	258 (16.9)	52	29.5 (22.5, 38.7)	2.07 (1.48, 2.88)		
GP registration at interview						
No	53 (3.9)	6	14.0 (5.8, 33.5)	Reference		
Yes	1402 (96.1)	173	10.1 (14.6, 19.7)	1.30 (0.54, 3.11)		0.55
Not born in UK: English as first language^g^						
No	308 (56.6)	28	7.8 (4.1, 12.0)	Reference		
Yes	235 (43.4)	25	7.4 (4.5, 12.0) 7.4 (4.5, 12.0)	0.98 (0.51, 1.9)		0.95
Total	1455 (100)	179	14.4 (12.4, 16.7)			

Rates of referral to psychological treatment per thousand-person years of observation with 95% confidence intervals (95% CI) are shown. Estimates for proportions, rates, and rate ratios account for non-response weights and household clustering. Unless otherwise stated, *p*-values refer to the null hypothesis of no association between the row variable and rate of IAPT referral

^a^Lifetime adverse life events comprised adulthood witnessed violence, being exposed to a war zone, being victim to a crime, injury with a weapon, or being attacked, adulthood separation, death of a loved one, serious accident/injury, and homelessness

^b^Common mental disorder was a categorical variable based on groupings of 0–11, 12–17, and 18 and above on the Comprehensive Interview Schedule, Revised (CIS-R)

^c^Drug use was based on responses to items for ingestion of cannabis, crack, cocaine, ecstasy, LSD, and heroin in the previous year

^d^Hazardous alcohol use was measured using the AUDIT scale, using a cut-off of 8 to identify hazardous alcohol use

^e^*p* value for linear association with number of lifetime adverse life events, *p* ≤ 0.01, with insufficient statistical evidence to reject linearity, *p* = 0.41

^f^*p* value for linear association with severity of CMD, *p* ≤ 0.01 with insufficient statistical evidence to reject linearity, *p* = 0.25

^g^As analysis of this variable was among migrants only, frequencies, percentages, rates, and ratios for this variable are for migrants only. Therefore, frequencies do not add up to 1455

### The rate of psychological treatment use

The overall rate of psychological treatment use was 14.4 cases per thousand person-years (cases/1000 pyrs, 95% CI 12.4, 16.7). For migration status, the rate of use of psychological treatment ranged from 21.1 cases/1000 pyrs in those residing in the UK for 10–20 years, to 7.1 cases/1000 pyrs in those residing in the UK for less than 10 years (rate ratio 0.4, 95% CI 0.2, 0.7, *p*-value for overall association 0.01). Black African participants experienced the lowest rate of psychological treatment use (12.2 cases/1000 pyrs), and the other ethnic group experienced the highest rate (21.7 cases/1000 pyrs), although rate ratio comparisons did not suggest sufficiently strong statistical evidence for this (*p* = 0.39). Survey respondents residing in Lambeth at the time of interview experienced a lower rate of use of psychological treatment compared to Southwark residents (RR 0.7, 95% CI 0.5, 0.9). Unemployed participants experienced a rate of 26.8 cases/1000 pyrs, compared to a rate in the employed of 14.7 cases/1000 pyrs (RR 1.8, 95% CI 1.2, 2.9). For educational attainment, rates ranged from 16.1 cases/1000 pyrs in those with no qualifications, to 20.1/1000 pyrs in those with GCSE level attainment. Female gender, poor physical functioning, severity of CMD symptoms, childhood abuse, the number of lifetime adverse life events, were statistically associated with a higher rate of psychological treatment use. Associations between migration status and other variables in the analysis are presented in Supplementary Table 1 for descriptive purposes. Migration status was associated with ethnic group in our sample (*p* ≤ 0.01)—for example, 4.6% (40) Black African participants were born in the UK, compared to 28.8% of those residing in the UK for less than 10 years. In categorical comparisons, migration status was also statistically associated with younger age, greater proportions employed, higher educational attainment, higher proportions of those married/cohabiting, lower drug use in the previous year, lower levels of hazardous alcohol use, and poor physical functioning (all *p* ≤ 0.01).

### Multivariable modelling

Unadjusted and fully adjusted estimates for association of migration, and ethnicity, with use of psychological treatment, are presented in Table 2. In unadjusted comparisons, participants residing in the UK for less than 10 years at interview experienced 0.41-fold lower rates of psychological treatment use compared to those who were born in the UK. There were very small differences made these estimates on adjustment for covariates, with the final rate ratio estimate of 0.4 (95% CI 0.2, 0.8). There was statistical evidence for an overall association between migration status and rate of psychological treatment use after all adjustments (*p* = 0.01).

**Table 2:** Multivariable model estimates for association (in the form of rate ratios, weighted for non-response and accounting for household clustering) of migration status, and ethnicity, with time to referral for psychological treatment

	Unadjusted RR (95%CI)	Model 1 RR (95%CI)	Model 5 RR (95%CI)	Final model *p*-values (Wald)
Migration status				
Born in the UK	Reference	Reference	Reference	
Longer than 20 years	1.0 (0.6, 1.7)	1.2 (0.7, 2.1)	1.2 (0.7, 2.1)	
10–20 years	1.3 (0.8, 2.2)	1.3 (0.8, 2.2)	1.3 (0.7, 2.3)	
Less than 10 years	0.4 (0.2, 0.8)	0.4 (0.2, 0.7)	0.4 (0.2, 0.8)	*0.01*
Ethnicity				
White	Reference	Reference	Reference	
Black Caribbean	0.7 (0.4, 1.4)	0.6 (0.3,1.3)	0.5 (0.2, 1.1)	
Black African	0.8 (0.4, 1.4)	0.7 (0.4, 1.2)	0.6 (0.3, 1.2)	
South Asian	0.7 (0.3, 1.7)	0.6 (0.2, 1.6)	0.7 (0.3, 1.7)	
Other	1.4 (0.8, 2.4)	1.3 (0.8, 2.2)	1.3 (0.7, 2.2)	0.14

All models are based on 1346 participants with complete data on all modelled variables. Models 2–5 are presented in Table S2 of the Supplementary Material

Italic value indicates the *p*-value was statistcally significant at the 0.05 level

Model 1 was adjusted for calendar period, age, gender, borough, employment, educational attainment, and marital status; model 2: further adjusted for CMD and poor physical functioning; model 3: further adjusted for number of childhood abuse and lifetime adverse life events; model 4: further adjusted for drug use and hazardous alcohol use; model 5: further adjusted for GP registration at interview. Full model estimates from model 5 are presented in Table 3

Although point estimates indicated lower rates of psychological treatment use among Black Caribbean, Black African, and South Asian respondents compared to White respondents, confidence intervals crossed null, indicating insufficient statistical evidence. Adjustments had limited influence on estimates. There remained no statistical evidence for association between ethnicity and psychological treatment use after all adjustments (*p* = 0.12). Estimates for association of migration status and ethnicity with psychological treatment use via GP referral, and psychological treatment use via self-referral were similar, although statistical evidence was stronger for lower rate in the most recent migrants for GP referrals (RR 0.3, 95% CI 0.1, 0.8), but not for self-referrals (RR 0.4, 95% CI 0.2, 1.0). Final estimates for migration and ethnicity, before accounting for secondary mental health service use as competing outcome, are presented in Table 3.

**Table 3:** Final Poisson regression model estimates for the rate of referral to psychological treatment based on 1346 participants, incorporating survey weights and accounting for household clustering

	Rate ratio	Lower 95% CI	Upper 95%CI
Migration status			
Born in the UK	Reference		
In the UK longer than 20 years	1.2	0.7	2.1
10–20 years	1.3	0.7	2.3
Less than 10 years	0.4	0.2	0.8
Ethnicity			
White	Reference		
Black Caribbean	0.5	0.2	1.1
Black African	0.6	0.3	1.2
South Asian	0.7	0.3	1.7
Other	1.3	0.7	2.2
Calendar period of observation			
2008–2009	Reference		
2010–2011	0.8	0.3	2.1
2012–2013	1.4	0.6	3.7
2014–2015	1.0	0.4	2.7
2016–2018	1.0	0.4	2.7
Age in years	1.0	1.0	1.0
Borough of residence			
Southwark	Reference		
Lambeth	0.7	0.5	0.9
Gender			
Male	Reference		
Female	1.7	1.2	2.4
Employment			
Employed	Reference		
Student	1.0	0.6	1.7
Unemployed	1.5	0.9	2.7
Other	1.2	0.8	2.0
Educational attainment			
No qualifications	Reference		
GCSE	1.4	0.7	2.6
A-level	1.6	0.8	3.0
Degree level or above	1.3	0.7	2.6
Marital status			
Single	Reference		
Married/cohabiting	1.0	0.7	1.6
Divorced/separated	1.4	0.8	2.5
Widowed	1.6	0.6	4.3
Number of lifetime adverse life events	1.1	1.0	1.2
Childhood abuse			
No	Reference		
Yes	1.2	0.8	1.8
CMD^a^	1.4	1.1	1.9
Poor physical functioning			
	Reference		
	1.2	0.8	1.9
Drug use			
No	Reference		
Yes	1.4	0.9	2.2
Hazardous alcohol use			
No	Reference		
Yes	0.7	0.5	1.1
GP registration			
No	Reference		
Yes	1.6	0.6	4.5

^a^CMD was included in models as described in Table 1, that is, with the coefficient reflecting an average relative increase in rate from no CMD (CISR score 0–11), to mild/moderate CMD (CISR score 12–17), to severe CMD (CISR score 18 and above)

In migrants-only analyses (Table 4), there was statistical evidence for a lower rate of psychological treatment use in the most recent migrants compared to those residing in the UK for longer than 20 years in unadjusted comparisons (RR 0.3, 95% CI 0.2, 0.7), which attenuated to 0.4 (95% CI 0.2, 1.2) on adjustment. There was limited impact on our estimates of accounting for migration for asylum/political reasons, or English as first language. There was statistical evidence for non-linearity in association with increasing years of residence (*p* = 0.02). Including referral to secondary mental health services as a competing outcome did not alter inferences (adjusted sub-hazard ratio (SHR) for psychological treatment use for migrants residing in the UK for less than 10 years compared to non-migrants: 0.4, 95% CI 0.2, 0.9, Table 5).

**Table 4:** Among migrants only, the association of years of residence with the rate of referral to psychological treatment, assessing non-linearity, asylum, and English language preference^a^

	*N* (%)	Unadjusted	Final model before adjustment for asylum/language	Further adjusting for asylum	Further adjusting for preferred language
Years of residence					
Longer than 20 years	164 (28.6)	Reference	Reference	Reference	Reference
10–20 years	135 (25.2)	1.1 (0.6, 2.1)	1.5 (0.7, 3.4)	1.4 (0.6, 3.5)	1.5 (0.6, 3.8)
Less than 10 years	244 (46.3)	0.3 (0.2, 0.7)	0.4 (0.2, 1.2)	0.4 (0.2, 1.1)	0.4 (0.2, 1.2)^b^
Ethnicity					
White	214 (39.2)	Reference	Reference	Reference	Reference
Black Caribbean	48 (8.3)	0.2 (0.1, 1.00)	0.1 (0.0, 0.8)	0.2 (0.0, 1.4)	0.2 (0.0, 1.1)
Black African	147 (27.4)	0.7 (0.4, 1.4)	0.6 (0.3, 1.3)	0.8 (0.4, 1.7)	0.8 (0.4, 1.7)
South Asian	33 (6.1)	0.6 (0.2, 2.0)	0.3 (0.1, 1.4)	0.3 (0.1, 1.3)	0.3 (0.1, 1.4)
Other	101 (19.0)	0.9 (0.4, 1.9)	1.0 (0.5, 2.3)	1.2 (0.5, 3.1)	1.3 (0.5, 3.3)
Migrated for asylum/political reasons					
No	503 (92.6)			Reference	Reference
Yes	40 (7.4)	–	–	0.5 (0.1, 1.6)	0.4 (0.1, 1.6)
English as first language					
No	308 (56.6)			–	Reference
Yes	235 (43.4)	–	–	–	1.4 (0.7, 2.8)

^a^Final model as per Table 3

^b^*p* for non-linearity = 0.02

**Table 5:** Association of migration status with rate of referral for psychological treatment accounting for referral to secondary mental health services as a competing risk, estimating sub-hazard ratios, SHRs, with 95% CI

	SHR	Lower 95% CI	Upper 95% CI
Migration status			
Born in the UK	Reference		
In the UK longer than 20 years	1.4	0.8	2.6
10–20 years	1.1	0.5	2.2
Less than 10 years	0.5	0.2	0.9
Ethnicity			
White	Reference		
Black Caribbean	0.7	0.3	1.6
Black African	0.7	0.4	1.4
South Asian	0.7	0.2	2.4
Other	0.9	0.5	1.8
Borough of residence			
Southwark	Reference		
Lambeth	0.5	0.3	0.7
Age	1.00	1.0	1.0
Gender			
Male	Reference		
Female	2.3	1.5	3.5
Employment status			
Employed	Reference		
Students	0.9	0.5	1.8
Unemployed	1.3	0.7	2.4
Other	0.8	0.4	1.6
Educational attainment			
No qualifications	Reference		
GCSE	1.3	0.6	3.1
A-level	1.7	0.7	4.0
Degree level or above	1.5	0.6	3.4
Marital status			
Single	Reference		
Married/cohabiting	1.5	0.9	2.5
Divorced/separated	1.4	0.7	2.7
Widowed	1.2	0.3	4.5
Number of lifetime adverse life events	1.0	0.9	1.1
Childhood abuse			
No	Reference		
Yes	1.1	0.7	1.9
CMD	1.1	0.8	1.5
Poor physical functioning			
No	Reference		
Yes	1.5	0.8	2.6
Drug use			
No	Reference		
Yes	1.7	1.0	2.8
Hazardous alcohol use			
No	Reference		
Yes	0.6	0.3	1.0
GP registration			
No	Reference		
Yes	1.1	0.3	3.8

## Discussion

We found strong statistical evidence that migrants residing for less than 10 years in the UK were less likely to use psychological treatment through IAPT compared to those born in the UK, after accounting for demographic and socioeconomic indicators, childhood abuse, lifetime adverse life events, psychiatric/physical morbidity, GP registration, and the use of non-IAPT mental health services. This was also evident on consideration of both self-referral and GP-referrals separately. We found no strong influence of migrating for asylum/political reasons, or English proficiency, on our estimates of lower rate of psychological treatment use among migrants residing in the UK for less than 10 years. Our adjusted estimates also suggested lower rates of psychological treatment use in Black Caribbean, Black African, and South Asian minority ethnic groups, however there was limited statistical evidence for these differences.

We focused on disparities in the rate of psychological treatment use in a representative epidemiological sample with directly-measured information on use of IAPT-based psychological treatment (as opposed to self-reported data). In contrast to previous research, we accounted for a broad range of alternative explanations and assessed the impact of secondary mental health service use as a competing outcome, over a 10 year follow-up period. We carried out the study in a densely populated area with high levels of migration, affording sufficient numbers of participants to assess asylum/political migration and English language proficiency as explanations for differences in psychological treatment use within migrants.

Although we found statistical evidence for association of migration status with rate of psychological treatment use, estimates for ethnicity, and for duration of residence in migrants only, were statistically imprecise. It is possible to consider individuals as belonging to more complex categories than those defined by ethnic group or migration status, with inequalities only evident when such intersections are taken into account [18]. For example, there are important differences between migrants of different ethnic groups, not only in terms of their geographic origins, but also in reasons for migration (e.g., the high prevalence of economic reasons for migration among White migrants from the rest of Europe into the UK compared to migrants from elsewhere, as shown in Table S4). Self-ascribed ethnic categories may conflate populations with diverse arrays of cultural, religious, and historical affiliation and ethnic background [8, 10]. It is also possible that remaining variation in psychological treatment use could be related to other aspects of personal identity, e.g., sexual orientation and identity, or aspects of cultural or class identity we were unable to capture. This is important given previous research showing association between discriminatory experiences and CMD [19]—by leaving out some characteristics which in themselves could have been reasons for discrimination from our analyses, we may have been unable to capture the impact of discrimination as a driver for use of psychological treatment on our results. Information on discrimination, including anticipated discrimination in different settings, is available on a follow-up subset on SELCoH-1, and could be a focus for future work. Information on potential confounders was available from survey data collected at a single time point, limiting our ability to assess the impact of changing socioeconomic status on psychological treatment since migration. Our approach to accounting for CMD assumed no interaction between CMD and other psychiatric symptoms in relation to psychological treatment use, however public health burden of psychiatric symptoms in the general population may be driven in part by symptoms in combination [4]. Although we adjusted for the severity of CMD at the time of interview, it remains possible that lower psychological treatment use among the most recent migrants is explained by lower psychiatric morbidity compared to non-migrants, which we did not capture, because we did not adjust for the impact of other psychiatric symptoms. We used information on migration for asylum/political reasons in this paper, however we were not able assess legal status among migrants in the sample, which could exert a greater impact on CMD and help-seeking [6]. Our assessment of reasons for migration was based on self-report, which may have resulted in incomplete information on this variable. Our study objectives focussed on psychological treatment, and we did not look for differences in use of other interventions, such as psychotropic medication use, in this study. Our study was set in two boroughs in a densely populated inner city urban area with high levels of psychiatric morbidity relative to the rest of the UK. While our results may be generalizable to other urban communities with significant inward number of migrants in England, wider generalisation of our findings is not possible.

Previous research on use of psychological treatment specifically is limited, and has been carried out predominantly in American settings, with limited assessment of migration [15]. For example, Mojtabai et al. used data from the National Ambulatory Medical Care Survey to examine national trends in psychotherapy, finding similar odds of psychotherapy visits by ethnic group [26]. Similarly, Chen and Rizzo found no significant differences in psychotherapy use between White, African American, and Latino responders to the Medicaid Expenditure Panel Survey [11]. Olfson and colleagues analysed Medicare data to estimate rates of psychotherapy use among White patients to be more than twice that of Black or Hispanic individuals [28]. These studies did not collect information on migration. There are also clear differences between the USA healthcare contexts in which these studies were done, and the UK, limiting comparison with our results, particularly given interplay between migration and ethnic group in diverse geographic contexts.

Newly arrived migrants appear to use less primary, outpatient, and inpatient care compared to UK born individuals [30], however the extent of any such disparity for psychological treatment has not yet been examined. A variety of factors could explain lower use of psychological treatment in recent migrants observed in the present study. Lower psychological treatment use among the most recent migrants to the UK, compared to more long-standing migrants, could be because more recent migrants may have had less opportunity to build up knowledge and awareness of the availability of psychological treatment services. Further research directly assessing awareness of availability of local services is required to address this explanation. Migration, particularly for reasons of asylum, may be accompanied by mistrust of statutory services and more limited help-seeking for health problems [24, 25]. Our adjustment for a self-reported item for GP registration only indirectly accounts for trust in health services as a whole. Previous work has also suggested that more recent migrants are less likely to be registered with a GP, which could also explain lower levels of IAPT use [17], although we did not find evidence to support this. Use of psychological treatment may be influenced by cultural factors such as language proficiency in communicating psychological needs to clinicians, which may be limited, where there is a language barrier. This can be exacerbated by a lack of available translation, or use of inappropriate translators, and may be more pronounced in clinical situations, where conversation is lengthy and detailed, such as psychological treatment. While we adjusted for English as first language, to approximate language proficiency, this was a relatively crude measure. It remains possible that language differences affecting the most recent migrants partly explained lower use of psychological treatment. Information on markers of acculturation and cultural assimilation were unavailable in this study. Further research may be helpful in examining how migrants experience inter-related processes of acculturation, socioeconomic stratification, and help-seeking for CMD, to improve understanding of the differences reported in this paper.

## Conclusions

Recent migrants may be an underserved group for use of psychological treatment. Public information on IAPT should take account of linguistic diversity and cultural exclusion experienced by the most recent migrants to the UK. Further research is needed to examine individual and structural factors which may explain migration-related differences in psychological treatment use reported here. For example, culturally-adapted interventions could improve acceptability of psychological treatment, reducing disparities in use of psychological treatment.

## Supplementary information

Below is the link to the electronic supplementary material.

Supplementary file1 (DOCX 42 KB)

Supplementary file2 (DOCX 33 KB)

## Data Availability

The data that support the findings of this study are available for academic research purposes from the SELCoH study team via application to the SELCoH oversight committee.

## References

[1] Altman DG, Gore SM, Gardner MJ, Pocock SJ (1983). Statistical guidelines for contributors to medical journals. Br Med J (Clin Res Ed).

[2] Bauer AM, Alegría M (2010). Impact of patient language proficiency and interpreter service use on the quality of psychiatric care: a systematic review. Psychiatr Serv.

[3] Bear L, Finer R, Guo S, Lau AS (2014). Building the gateway to success: an appraisal of progress in reaching underserved families and reducing racial disparities in school-based mental health. Psychol Serv.

[4] Bhavsar V, Dorrington S, Morgan C, Hatch S, McGuire P, Fusar-Poli P (2021). Psychotic experiences, psychiatric comorbidity and mental health need in the general population: a cross-sectional and cohort study in Southeast London. Psychol Med.

[5] Bhavsar V, Maccabe JH, Hatch SL, Hotopf M, Boydell J, Mcguire P (2017). Subclinical psychotic experiences and subsequent contact with mental health services. Br J Psychiatry Open.

[6] Bhugra D (2004). Migration and mental health. Acta Psychiatr Scand.

[7] Bhui K, Stansfeld S, Hull S, Priebe S, Mole F, Feder G (2003). Ethnic variations in pathways to and use of specialist mental health services in the UK. Syst Rev.

[8] Braun L, Fausto-Sterling A, Fullwiley D, Hammonds EM, Nelson A, Quivers W, Reverby SM, Shields AE (2007). Racial categories in medical practice: how useful are they?. PLoS Med.

[9] Brown J, Ferner H, Wingrove J, Aschan L, Hatch S, Hotopf M (2014). How equitable are psychological therapy services in South East London now? A comparison of referrals to a new psychological therapy service with participants in a psychiatric morbidity survey in the same London borough. Soc Psychiatry Psychiatr Epidemiol.

[10] Burchard EG, Ziv E, Coyle N, Gomez SL, Tang H, Karter AJ, Mountain JL, Pérez-stable EJ, Sheppard D, Risch N (2003). The importance of race and ethnic background in biomedical research and clinical practice. N Engl J Med.

[11] Chen J, Rizzo J (2010). Racial and ethnic disparities in use of psychotherapy: evidence from US national survey data. Psychiatr Serv.

[12] Clark C, Pike C, Mcmanus S, Harris J, Bebbington P, Brugha T, Jenkins R, Meltzer H, Weich S, Stansfeld S (2012). The contribution of work and non-work stressors to common mental disorders in the 2007 Adult Psychiatric Morbidity Survey. Psychol Med.

[13] Clark DM (2011). Implementing NICE guidelines for the psychological treatment of depression and anxiety disorders: the IAPT experience. Int Rev Psychiatry.

[14] Clark DM, Layard R, Smithies R, Richards DA, Suckling R, Wright B (2009). Improving access to psychological therapy: initial evaluation of two UK demonstration sites. Behav Res Ther.

[15] Cooper C, Spiers N, Livingston G, Jenkins R, Meltzer H, Brugha T, Mcmanus S, Weich S, Bebbington P (2013). Ethnic inequalities in the use of health services for common mental disorders in England. Soc Psychiatry Psychiatr Epidemiol.

[16] Eaton WW, Martins SS, Nestadt G, Bienvenu OJ, Clarke D, Alexandre P (2008). The burden of mental disorders. Epidemiol Rev.

[17] Gazard B, Frissa S, Nellums L, Hotopf M, Hatch SL (2015). Challenges in researching migration status, health and health service use: an intersectional analysis of a South London community. Ethn Health.

[18] Goodwin L, Gazard B, Aschan L, Maccrimmon S, Hotopf M, Hatch SL (2018). Taking an intersectional approach to define latent classes of socioeconomic status, ethnicity and migration status for psychiatric epidemiological research. Epidemiol Psychiatr Sci.

[19] Hatch, S., Gazard, B., Williams, D., Frissa, S., Goodwin, L., Hotopf, M. & Team, S. S (2016). Discrimination and common mental disorder among migrant and ethnic groups: findings from a South East London Community sample. Soc Psychiatry Psychiatr Epidemiol.

[20] Hatch SL, Frissa S, Verdecchia M, Stewart R, Fear NT, Reichenberg A, Morgan C, Kankulu B, Clark J, Gazard B (2011). Identifying socio-demographic and socioeconomic determinants of health inequalities in a diverse London community: the South East London Community Health (SELCoH) study. BMC Public Health.

[21] Hepgul N, King S, Amarasinghe M, Breen G, Grant N, Grey N, Hotopf M, Moran P, Pariante CM, Tylee A, Wingrove J, Young AH, Cleare AJ (2016). Clinical characteristics of patients assessed within an Improving Access to Psychological Therapies (IAPT) service: results from a naturalistic cohort study (Predicting Outcome Following Psychological Therapy; PROMPT). BMC Psychiatry.

[22] Layard R (2006). The case for psychological treatment centres. BMJ.

[23] Lewis G, Pelosi A (1990). Manual of the revised clinical interview schedule (CIS-R).

[24] Loewenthal D, Mohamed A, Mukhopadhyay S, Ganesh K, Thomas R (2012). Reducing the barriers to accessing psychological therapies for Bengali, Urdu, Tamil and Somali communities in the UK: some implications for training, policy and practice. Br J Guid Couns.

[25] Majumder P, O’Reilly M, Karim K, Vostanis P (2015). ‘This doctor, I not trust him, I’m not safe’: the perceptions of mental health and services by unaccompanied refugee adolescents. Int J Soc Psychiatry.

[26] Mojtabai R, Olfson M (2008). National trends in psychotherapy by office-based psychiatrists. Arch Gen Psychiatry.

[27] Ohtani A, Suzuki T, Takeuchi H, Uchida H (2015). Language barriers and access to psychiatric care: a systematic review. Psychiatr Serv.

[28] Olfson M, Marcus SC (2010). National trends in outpatient psychotherapy. Am J Psychiatry.

[29] Office for National Statistics (2011) Table CT0787: ethnic group by year of arrival in the UK. ONS, London. https://www.ons.gov.uk/peoplepopulationandcommunity/culturalidentity/ethnicity/adhocs/008325ct07872011censusethnicgroupbyyearofarrivalintheuknationaltolocalauthority

[30] Saunders CL, Steventon A, Janta B, Stafford M, Sinnott C, Allen L, Deeny SR (2021). Healthcare utilization among migrants to the UK: cross-sectional analysis of two national surveys. J Health Serv Res Policy.

[31] Saunders JB, Aasland OG, Babor TF, De La Fuente JR, Grant M (1993). Development of the alcohol use disorders identification test (AUDIT): WHO collaborative project on early detection of persons with harmful alcohol consumption-II. Addiction.

[32] Sentell T, Shumway M, Snowden L (2007). Access to mental health treatment by English language proficiency and race/ethnicity. J Gen Intern Med.

[33] Stagg HR, Jones J, Bickler G, Abubakar I (2012) Poor uptake of primary healthcare registration among recent entrants to the UK: a retrospective cohort study. BMJ Open 2(4) 10.1136/bmjopen-2012-001453 PMC4400681 22869094

[34] StataCorp (2015) Stata statistical software: release 14. StataCorp LP, College Station, TX

[35] Steel Z, Marnane C, Iranpour C, Chey T, Jackson JW, Patel V, Silove D (2014). The global prevalence of common mental disorders: a systematic review and meta-analysis 1980–2013. Int J Epidemiol.

[36] Ware JE, Kosinski M (2001) SF-36 physical & mental health summary scales: a manual for users of version 1. Quality Metric Incorporated

