# Supplementary material for: The association of migration and ethnicity with use of the Improving Access to Psychological Treatment (IAPT) programme: a general population cohort study
Source: Soc Psychiatry Psychiatr Epidemiol. 2021 Feb 16;56(11):1943–56. doi: 10.1007/s00127-021-02035-7 (PMC8519879; doi:10.1007/s00127-021-02035-7)
Supplement: Supplementary file 1 — Supplementary file1 (DOCX 42 KB) [file 127_2021_2035_MOESM1_ESM.docx]

| Table S1. Migration status at survey interview, described by characteristics analysed in this study, on 1455 survey participants consenting to data linkage. Column percentages are shown, weighted for survey non-response and household clustering. Chi-squared (*X^2^)* comparisons are based on complete data. | | | | | | | | | | | | |
| --- | --- | --- | --- | --- | --- | --- | --- | --- | --- | --- | --- | --- |
|  | | | | Born in the UK | | Not born in UK: years of UK residence | | | | | | *X^2^*, degrees of freedom, p-value |
|  |  |  |  |  |  | Greater than 20 years | | 10-20 years | | Less than 10 years in UK | |  |
|  | | | | N | (%) | N | (%) | N | (%) | N | (%) |  |
| Referral to IAPT | | | |  |  |  |  |  |  |  |  |  |
| No IAPT referral | | |  | 788 | (86.6) | 149 | (91.0) | 85 | (87.0) | 232 | (95.3) |  |
| IAPT referral | | GP-referral | | 68 | (7.3) | 9 | (5.3) | 8 | (8.3) | 6 | (2.4) |  |
|  |  | Self-referral | | 56 | (6.1) | 6 | (3.7) | 5 | (4.7) | 6 | (2.2) | 16.2, 6, 0.01 |
| Ethnicity | White | | | 720 | (78.1) | 58 | (35.6) | 59 | (43.7) | 97 | (39.0) | 352.9, 12, <0.01 |
| Black Caribbean | | | | 68 | (7.7) | 30 | (17.5) | 4 | (3.0) | 14 | (5.5) |  |
| Black African | | | | 40 | (4.6) | 30 | (18.4) | 47 | (34.9) | 70 | (28.8) |  |
| South Asian | | | | 15 | (1.8) | 19 | (11.7) | 7 | (4.9) | 7 | (3.2) |  |
| Other | | | | 67 | (7.5) | 27 | (16.8) | 18 | (13.4) | 56 | (23.4) |  |
| Missing | | | | 2 | (0.2) | 0 | (0.0) | 0 | (0.0) | 0 | (0.0) |  |
| Age at interview | | | |  |  |  |  |  |  |  |  | 271.2, 6, <0.01 |
| 16-34 | | | | 397 | (46.4) | 8 | (6.1) | 45 | (35.4) | 172 | (71.8) |  |
| 34-54 | | | | 317 | (33.9) | 67 | (41.1) | 82 | (58.8) | 64 | (25.3) |  |
| 55- | | | | 198 | (19.7) | 89 | (52.7) | 8 | (5.8) | 8 | (2.9) |  |
| Gender | | | |  |  |  |  |  |  |  |  | 4.3, 3, 0.23 |
| Male | | | | 411 | (49.4) | 72 | (47.4) | 58 | (47.3) | 92 | (42.2) |  |
| Female | | | | 501 | (50.6) | 92 | (52.6) | 77 | (52.7) | 152 | (57.8) |  |
| Borough of residence | | | |  |  |  |  |  |  |  |  | 1.71, 3, 0.64 |
| Southwark | | | | 458 | (50.0) | 76 | (46.4) | 72 | (53.8) | 126 | (52.4) |  |
| Lambeth | | | | 454 | (50.0) | 88 | (53.6) | 63 | (46.2) | 118 | (47.6) |  |
| Employment | | | |  |  |  |  |  |  |  |  | 105.6, 9, <0.01 |
| Employed | | | | 490 | (53.8) | 69 | (42.9) | 81 | (60.0) | 144 | (59.0) |  |
| Students | | | | 141 | (16.9) | 5 | (3.8) | 15 | (12.0) | 48 | (20.6) |  |
| Unemployed | | | | 89 | (9.9) | 10 | (6.0) | 19 | (14.3) | 26 | (10.5) |  |
| Other | | | | 189 | (19.0) | 79 | (46.5) | 20 | (13.8) | 24 | (9.1) |  |
| Missing | | | | 3 | (0.3) | 1 | (0.7) | 0 | (0.0) | 2 | (0.8) |  |
| Educational attainment | | | |  |  |  |  |  |  |  |  | 60.1, 9, <0.01 |
| No qualification | | | | 124 | (12.8) | 44 | (26.1) | 13 | (9.5) | 15 | (6.1) |  |
| GCSE | | | | 206 | (22.7) | 33 | (19.7) | 24 | (18.3) | 36 | (15.0) |  |
| A-level | | | | 197 | (22.4) | 36 | (22.3) | 44 | (33.3) | 77 | (31.2) |  |
| Degree level or above | | | | 378 | (41.4) | 47 | (29.5) | 52 | (37.6) | 114 | (46.8) |  |
| Missing | | | | 7 | (0.7) | 4 | (0.2) | 2 | (1.0) | 2 | (0.8) |  |
| Marital status | | | |  |  |  |  |  |  |  |  | 86.0, 9, <0.01 |
| Single | | | | 390 | (44.6) | 24 | (15.7) | 44 | (34.5) | 100 | (42.4) |  |
| Married/cohabiting | | | | 411 | (44.3) | 89 | (54.6) | 74 | (53.8) | 118 | (47.6) |  |
| Divorced/separated | | | | 85 | (8.7) | 35 | (20.6) | 17 | (11.7) | 24 | (9.2) |  |
| Widowed | | | | 26 | (2.5) | 16 | (9.1) | 0 | (0.0) | 2 | (0.7) |  |
| Number of lifetime adverse life events | | | | | | | |  |  |  |  | 11.5, 6, 0.07 |
| 0-2 | | | | 453 | (49.6) | 81 | (49.3) | 70 | (51.6) | 146 | (59.7) |  |
| 3-5 | | | | 392 | (43.0) | 66 | (40.5) | 54 | (39.9) | 87 | (35.7) |  |
| 6-8 | | | | 67 | (7.4) | 17 | (10.3) | 11 | (8.5) | 11 | (4.6) |  |
| Childhood abuse | | | |  |  |  |  |  |  |  |  | 1.1, 3, 0.77 |
| No | | | | 671 | (73.2) | 112 | (68.4) | 96 | (69.9) | 179 | (73.1) |  |
| Yes | | | | 237 | (26.4) | 48 | (29.1) | 36 | (27.8) | 63 | (26.2) |  |
| Missing | | | | 4 | (0.4) | 4 | (2.4) | 3 | (2.3) | 2 | (0.7) |  |
| Common mental disorder (CIS-R score) | | | | |  |  |  |  |  |  |  | 8.3, 6, 0.22 |
| 0-12 | | | | 684 | (75.4) | 120 | (73.8) | 101 | (75.2) | 197 | (81.2) |  |
| 12-18 | | | | 11 | (12.1) | 19 | (11.5) | 22 | (16.1) | 22 | (9.0) |  |
| Over 18 | | | | 114 | (12.2) | 25 | (14.7) | 12 | (8.7) | 25 | (9.7) |  |
| Missing | | | | 3 | (0.3) | 0 | (0.0) | 0 | (0.0) | 0 | (0.0) |  |
| Drug use | | | |  |  |  |  |  |  |  |  | 31.2, 3, <0.01 |
| No | | | | 670 | (72.0) | 145 | (87.8) | 113 | (82.6) | 204 | (83.3) |  |
| Yes | | | | 241 | (27.9) | 16 | (10.4) | 22 | (17.4) | 40 | (16.7) |  |
| Missing | | | | 1 | (0.1) | 3 | (1.8) | 0 | (0.0) | 0 | (0.0) |  |
| Hazardous alcohol use | | | |  |  |  |  |  |  |  |  | 40.4, 3, <0.01 |
| No | | | | 676 | (73.1) | 138 | (83.3) | 111 | (81.5) | 221 | (90.2) |  |
| Yes | | | | 234 | (26.7) | 21 | (13.8) | 23 | (17.8) | 22 | (9.4) |  |
| Missing | | | | 2 | (0.2) | 5 | (2.9) | 1 | (0.7) | 1 | (0.4) |  |
| Poor physical functioning | | | |  |  |  |  |  |  |  |  | 33.8, 3,  <0.01 |
| No | | | | 749 | (83.9) | 109 | (67.4) | 115 | (85.9) | 214 | (88.2) |  |
| Yes | | | | 154 | (16.1) | 55 | (32.6) | 20 | (14.1) | 30 | (11.8) |  |
| GP registration at interview | | | | | | | | | | | | 21.0, 3,  <0.01 |
| No | | | | 26 | (3.1) | 4 | (2.5) | 2 | (1.4) | 20 | (8.8) |  |
| Yes | | | | 877 | (96.9) | 160 | (97.5) | 133 | (98.6) | 223 | (91.2) |  |
| In migrants only | | | | | | | | | | | | |
| Migrated for asylum/political reasons | | | | | | |  |  |  |  |  | 3.9, 2,  0.15 |
| No | | | | - | - | 155 | (94.4) | 120 | (88.9) | 228 | (93.5) |  |
| Yes | | | | - | - | 9 | (5.6) | 15 | (11.1) | 16 | (6.5) |  |
| English as first language | | | | - | - |  |  |  |  |  |  | 14.3, 2,  0.01 |
| No | | | | - | - | 73 | (44.1) | 83 | (60.9) | 152 | (62.0) |  |
| Yes | | | | - | - | 91 | (55.9) | 52 | (39.1) | 92 | (38.0) |  |

| Table S2. Partially- and fully-adjusted estimates for association (in the form of rate ratios, weighted for non-response and accounting for household clustering) of migration status, and ethnicity, with time to referral for psychological treatment. Coefficients for migration and ethnicity were estimated in the same model. All models are based on 1346 participants with complete data on all modelled variables. | | | | | | | |
| --- | --- | --- | --- | --- | --- | --- | --- |
|  | Unadjusted  RR (95%CI) | Model 1  RR (95%CI) | Model 2  RR (95%CI) | Model 3  RR (95%CI) | Model 4  RR (95%CI) | Model 5  RR (95%CI) | Final model p-values (Wald) |
| Migration status |  |  |  |  |  |  |  |
| Born in the UK | Reference | Reference | Reference | Reference | Reference | Reference |  |
| Longer than 20 years | 1.0(0.6, 1.7) | 1.2(0.7, 2.1) | 1.2(0.7, 2.2) | 1.1(0.6, 2.0) | 1.1(0.6, 2.0) | 1.2(0.7, 2.1) |  |
| 10-20 years | 1.3(0.8, 2.2) | 1.3(0.8, 2.2) | 1.2(0.7, 2.1) | 1.3(0.7, 2.3) | 1.3(0.7, 2.3) | 1.3(0.7, 2.3) |  |
| Less than 10 years | 0.4(0.2, 0.8) | 0.4(0.2, 0.7) | 0.4(0.2, 0.7) | 0.4(0.2, 0.7) | 0.4(0.2, 0.7) | 0.4(0.2, 0.8) | *0.01* |
| Ethnicity |  |  |  |  |  |  |  |
| White | Reference | Reference | Reference | Reference | Reference | Reference |  |
| Black Caribbean | 0.7(0.4, 1.4) | 0.6(0.3,1.3) | 0.6(0.3, 1.2) | 0.6(0.3, 1.2) | 0.5(0.3, 1.1) | 0.5(0.2, 1.1) |  |
| Black African | 0.8(0.4, 1.4) | 0.7(0.4, 1.2) | 0.7(0.4, 1.2) | 0.6(0.3, 1.2) | 0.6(0.3, 1.2) | 0.6(0.3, 1.2) |  |
| South Asian | 0.7(0.3, 1.7) | 0.6(0.2, 1.6) | 0.6(0.2, 1.5) | 0.6(0.2, 1.7) | 0.7(0.3, 1.7) | 0.7(0.3, 1.7) |  |
| Other | 1.4(0.8, 2.4) | 1.3(0.8, 2.2) | 1.2(0.7, 2.1) | 1.3(0.7, 2.2) | 1.3(0.7, 2.2) | 1.3(0.7, 2.2) | 0.14 |
| Model 1 was adjusted for calendar period, age, gender, borough, employment, educational attainment, and marital status; Model 2: Further adjusted for CMD and poor physical functioning ; Model 3: Further adjusted for number of childhood abuse and lifetime adverse life events; Model 4: Further adjusted for drug use and hazardous alcohol use; Model 5: Further adjusted for GP registration at interview. | | | | | | | |

| Table S3. Partially- and fully-adjusted estimates for association (in the form of rate ratios, weighted for non-response and accounting for household clustering) of migration status, and ethnicity, with time to self-referral for psychological treatment, and GP referral for psychological treatment. Coefficients for migration and ethnicity were estimated in the same model. All models are based on 1346 participants with complete data on all modelled variables. | | | | | | | |
| --- | --- | --- | --- | --- | --- | --- | --- |
|  | Unadjusted  RR (95%CI) | Model 1  RR (95%CI) | Model 2  RR (95%CI) | Model 3  RR (95%CI) | Model 4  RR (95%CI) | Model 5  RR (95%CI) | Final model p-values (Wald) |
| Self referral |  |  |  |  |  |  |  |
| Migration status |  |  |  |  |  |  |  |
| Born in the UK | Reference | Reference | Reference | Reference | Reference | Reference |  |
| Longer than 20 years | 0.8(0.3,2.0) | 0.8(0.3, 2.2) | 0.8(0.3, 2.3) | 0.8(0.3, 2.2) | 0.8(0.3, 2.2) | 0.8(0.3, 2.3) |  |
| 10-20 years | 1.4(0.6,3.1) | 1.3(0.5, 3.0) | 1.2(0.5,3.0) | 1.3(0.5, 3.1) | 1.3(0.5, 3.2) | 1.3(0.5, 3.2) |  |
| Less than 10 years | 0.4(0.2, 1.2) | 0.4(0.1, 1.0) | 0.4(0.1, 1.0) | 0.4(0.1, 1.0) | 0.4(0.1, 1.0) | 0.4(0.2, 1.0) | 0.17 |
| Ethnicity |  |  |  |  |  |  |  |
| White | Reference | Reference | Reference | Reference | Reference | Reference |  |
| Black Caribbean | 0.8(0.3,1.9) | 0.6(0.2,1.6) | 0.6(0.2, 1.6) | 0.6(0.2, 1.6) | 0.6(0.2, 1.6) | 0.6(0.2, 1.6) |  |
| Black African | 0.4(0.1, 1.4) | 0.4(0.1, 1.4) | 0.4(0.1, 1.4) | 0.4(0.1, 1.4) | 0.4(0.1, 1.4) | 0.4(0.1, 1.4) |  |
| South Asian | 0.4(0.1, 3.4) | 0.4(0.0,3.2) | 0.4(0.1, 3.3) | 0.4(0.1, 3.3) | 0.4(0.1, 3.3) | 0.4(0.1, 3.3) |  |
| Other | 0.6(0.2, 1.6) | 0.5(0.2, 1.4) | 0.5(0.2, 1.4) | 0.6(0.2, 1.5) | 0.6(0.2, 1.5) | 0.6(0.2, 1.5) | 0.42 |
| GP referral |  |  |  |  |  |  |  |
| Migration status |  |  |  |  |  |  |  |
| Born in the UK | Reference | Reference | Reference | Reference | Reference | Reference |  |
| Longer than 20 years | 0.7(0.3, 1.7) | 1.0(0.5, 2.3) | 0.9(0.4, 2.2) | 0.9(0.4, 2.0) | 0.9(0.4, 2.0) | 0.9(0.4,2.1) |  |
| 10-20 years | 1.1(0.5, 2.3) | 1.0(0.4, 2.4) | 1.0(0.4, 2.4) | 1.1(0.5, 2.5) | 1.1(0.5, 2.4) | 1.1(0.5,2.5) |  |
| Less than 10 years | 0.3(0.1, 0.8) | 0.3(0.1, 0.8) | 0.3(0.1, 0.8) | 0.3(0.1, 0.8) | 0.3(0.1,0.8) | 0.3(0.1,0.8) | 0.10 |
| Ethnicity |  |  |  |  |  |  |  |
| White | Reference | Reference | Reference | Reference | Reference | Reference |  |
| Black Caribbean | 0.5(0.2, 1.7) | 0.4(0.1, 1.4) | 0.4(0.1, 1.3) | 0.4(0.1, 1.4) | 0.3(0.1, 1.3) | 0.3(0.1, 1.4) |  |
| Black African | 1.5(0.7, 3.2) | 1.1(0.5, 2.3) | 1.0(0.5, 2.2) | 1.0(0.4, 2.2) | 1.0(0.4, 2.2) | 0.9(0.4,2.2) |  |
| South Asian | 0.9(0.2, 3.3) | 0.7(0.2, 2.8) | 0.9(0.2, 3.8) | 0.9(0.2, 3.7) | 1.0(0.3,3.9) | 1.0(0.2,3.9) |  |
| Other | 2.3(0.1, 4.8) | 1.9(0.9, 3.9) | 1.9(0.9, 4.0) | 2.2(1.0, 4.6) | 2.1(1.0, 4.6) | 2.1(0.9, 4.6) | 0.11 |
| Model 1 was adjusted for calendar period, age, gender, borough, employment, educational attainment, and marital status; Model 2: Further adjusted for CMD and poor physical functioning ; Model 3: Further adjusted for number of childhood abuse and lifetime adverse life events; Model 4: Further adjusted for drug use and hazardous alcohol use; Model 5: Further adjusted for GP registration at interview. | | | | | | | |

| Table S4. Continent of migration, and reasons for migration, among migrants in this survey who consented to linkage to hospital records (n=543). Column percentages are shown, weighted for survey non-response and household clustering. Chi-squared (*X^2^)* comparisons are based on complete data. | | | | | | | | | | | | |
| --- | --- | --- | --- | --- | --- | --- | --- | --- | --- | --- | --- | --- |
|  | White | | Black Caribbean | | Black African | | Asian | | Other | | | *X^2^*, degrees of freedom, p-value |
| Continent of migration | |  |  |  |  |  |  |  |  |  | 1.2x10^-3^ ,24, <0.01 | |
| Europe | 138 | (64.8) | 0 | (0.0) | 2 | (1.4) | 1 | (3.0) | 9 | (8.8) |  |  |
| Africa | 14 | (6.5) | 1 | (2.2) | 140 | (95.2) | 4 | (11.2) | 19 | (18.1) |  |  |
| Caribbean | 0 | (0.0) | 45 | (93.9) | 1 | (0.7) | 0 | (0.0) | 2 | (1.9) |  |  |
| Asia | 3 | (1.4) | 0 | (0.0) | 0 | (0.0) | 28 | (85.7) | 39 | (40.2) |  |  |
| Americas | 33 | (15.2) | 2 | (3.9) | 1 | (0.7) | 0 | (0.0) | 31 | (30.1) |  |  |
| Australasia/Oceania | 23 | (10.7) | 0 | (0.0) | 0 | (0.0) | 0 | (0.0) | 0 | (0.0) |  |  |
| Missing | 3 | (1.5) | 0 | (0.0) | 3 | (1.9) | 0 | (0.0) | 1 | (0.9) |  |  |
| Reasons for migration | |  |  |  |  |  |  |  |  |  |  | |
| Work/Study | 102 | (47.9) | 7 | (15.2) | 45 | (31.5) | 10 | (32.0) | 43 | (43.2) |  | |
| Family | 63 | (29.4) | 28 | (5.8) | 60 | (40.1) | 18 | (52.6) | 26 | (24.9) |  | |
| War/political unrest | 5 | (2.2) | 0 | (0.0) | 18 | (12.2) | 1 | (2.7) | 16 | (16.1) |  | |
| Better life | 5 | (2.4) | 6 | (12.6) | 4 | (2.9) | 0 | (0.0) | 3 | (3.0) |  | |
| Other | 23 | (10.4) | 5 | (10.6) | 12 | (8.1) | 1 | (3.3) | 9 | (8.8) |  | |
| Missing | 16 | (7.6) | 2 | (3.9) | 8 | (5.2) | 3 | (9.4) | 4 | (3.9) | 76.6, 20 <0.01 | |
| Total | 214 | (100.0) | 48 | (100.0) | 147 | (100.0) | 33 | (100.0) | 101 | (100.0) |  | |
